# Mastite granulomateuse idiopathique associant un érythème noueux: à propos d’un cas

**DOI:** 10.11604/pamj.2022.43.196.26754

**Published:** 2022-12-16

**Authors:** Alpha Boubacar Conte, Fatima Zohra Fdili Alaoui, Sofia Jayi, Hikmat Chaara, Moulay Abdelilah Melhouf

**Affiliations:** 1 Université Sidi Mohamed Ben Abdellah, Service de Gynécologie-Obstétrique II, Centre Hospitalier Universitaire Hassan II de Fès, Fès, Maroc

**Keywords:** Mastite granulomateuse idiopathique, corticothérapie, tumorectomie, cas clinique, Idiopathic granulomatous mastitis, corticotherapy, lumpectomy, case report

## Abstract

La mastite granulomateuse idiopathique (MGI) est une maladie inflammatoire bénigne chronique du sein qui peut mimer un cancer du sein. Elle est plus fréquente chez les jeunes femmes en âge de procréer et pose un problème diagnostic d´une tumeur inflammatoire du sein. Nous rapportons le cas d´une patiente de 26 ans qui a consulté pour la prise en charge d´un sein inflammatoire chez qui l´examen a trouvé une patiente apyrétique avec un sein gauche siège d´une masse lisse irrégulière œdématiée et indolore mesurant 4 cm surmontée par des croûtes et de multiples cicatrices de fistulisation laissant sourdre du pus à la pression avec une adénopathie axillaire homolatérale mobile. Le reste de l´examen physique a trouvé sur les 02 jambes des plaques érythémateuses inflammatoires. Une mammographie plus échographie mammaire ont été réalisées suivie d´une biopsie qui revenait en faveur d´une mastopathie fibreuse. La persistance de la symptomatologie a conduit à la réalisation d´une tumorectomie dont l´anapath était en faveur d´une mastite granulomateuse. La recherche étiologique n´a pas permis de retrouver une cause évidente. Une corticothérapie associée à une antibiothérapie a été instaurée avec régression complète de la symptomatologie au bout de deux mois.

## Abstract (en)

Idiopathic granulomatous mastitis (IMD) is a chronic benign inflammatory disease of the breast which can mimic breast cancer. It is more common in young women of childbearing age and poses a challenge in diagnosis of inflammatory breast tumor. We report the case of a 26-year-old female patient who presented with inflammatory breast. Clinical examination showed apyretic patient with edematized and painless irregular smooth mass in the left breast measuring 4 cm surmounted by crusts and multiples fistulizing scars discharging pus by applying pressure, with mobile ipsilateral axillary adenopathy. The remainder of the physical examination showed inflammatory erythematous patches on both legs. A mammogram plus breast ultrasound was performed followed by biopsy that objectified fibrous mastopathy. Lumpectomy was performed due to the persistence of symptoms. Anatomopathological examination objectified granulomatous mastitis. Etiological research didn’t identify a clear cause. Corticotherapy associated with antibiotic therapy was started with complete regression of symptoms after two months.

## Introduction

La mastite granulomateuse idiopathique (MGI) est une maladie inflammatoire bénigne chronique du sein qui peut mimer un cancer du sein [1]. C´est une inflammation du sein d´origine inconnue qui doit être distinguée des tumeurs et des infections mammaires, y compris la tuberculose [2]. Sa symptomatologie est non spécifique et le diagnostic est souvent non évident. C´est une entité non bien connue des cliniciens et des radiologues [3]. Nous en rapportons un cas survenu chez une patiente âgée de 27 ans qui s´est manifesté par un sein inflammatoire avec de multiples fistulisations et des érythèmes noueux aux membres inférieurs.

## Patient et observation

**Information de la patiente**: patiente âgée de 27 ans, habitant en milieu rural qui a consulté pour la prise en charge d´un sein gauche inflammatoire. Elle est paucipare allaitante depuis 7 mois et sans antécédents particuliers. Le début de sa symptomatologie remonterait à 4 mois avant sa consultation et serait marqué par la survenue de façon récurrente d´écoulement mamelonnaire purulent associé à une modification cutanée à type de placards inflammatoires et de multiples fistulisation du sein ayant motivé une application locale d´herbicide. Vu la persistance de la symptomatologie et l´apparition des lésions nodulaires érythémateuses rougeâtres aux membres, la patiente a décidé de consulter dans notre structure pour une prise en charge.

**Figure 1:**
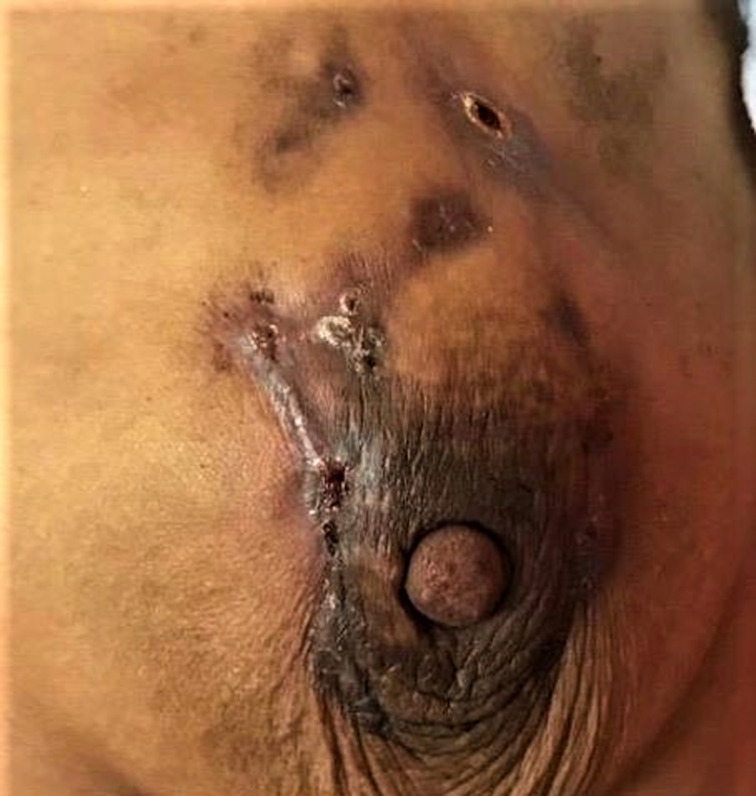
sein avec de multiples cicatrices de fistulisation

**Figure 2:**
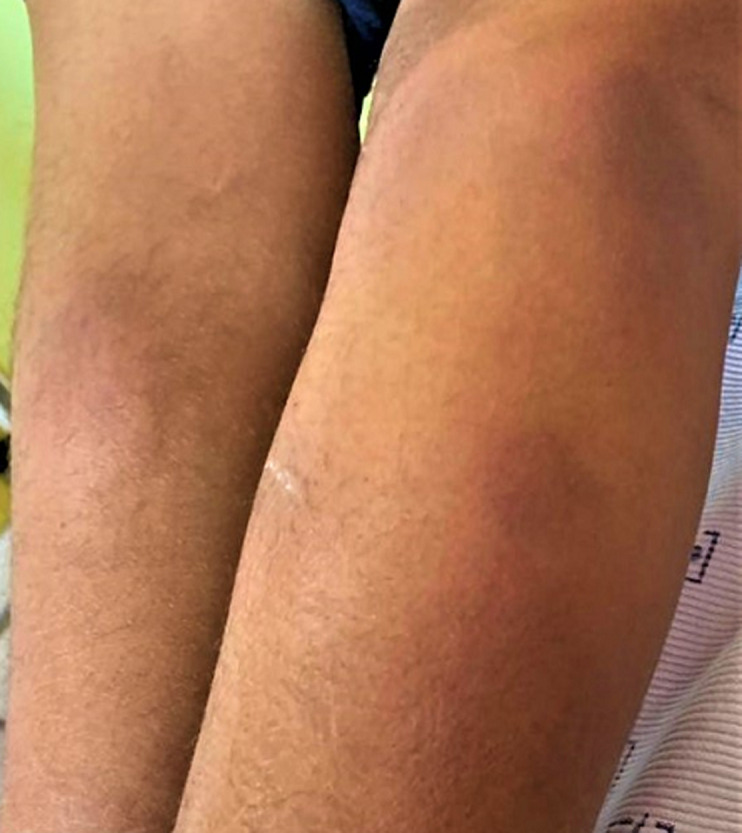
plaques érythémateuses inflammatoires sur les 2 jambes

**Résultat clinique**: l´examen général avait trouvé une patiente apyrétique dans un bon état général. L´examen des seins a trouvé des seins asymétriques avec un sein gauche inflammatoire siège d´une masse lisse irrégulière œdématiée et indolore mesurant 4 cm surmontées par des croûtes et de multiples cicatrices de fistulisation (Figure 1) laissant sourdre du pus à la pression avec une adénopathie axillaire homolatérale mobile. Le sein controlatéral était sans particularité. Le reste de l´examen physique a trouvé sur les 02 jambes des plaques érythémateuses inflammatoire rougeâtres, chaude et ovale (Figure 2).

**Figure 3:**
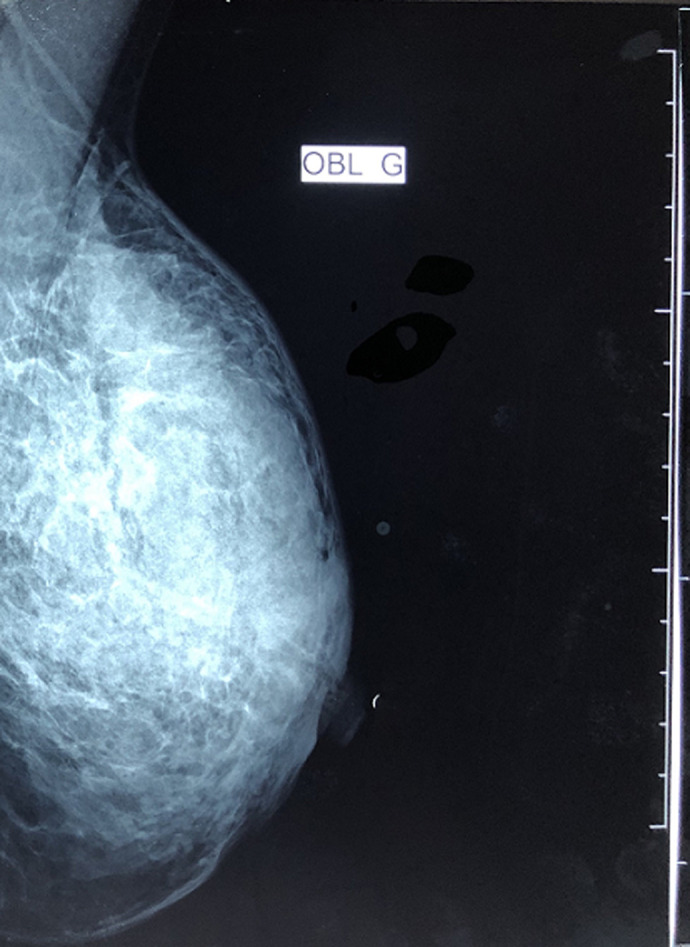
opacités à contours mal définis des quadrant supéro externe et jonction des quadrants supérieurs

**Démarche diagnostique**: une mammographie réalisée trouvait une opacité à contours mal définis (Figure 3) intéressant le quadrant supéro externe (QSE) et la jonction des quadrants supérieurs (JQS) du sein gauche associée à un épaississement du revêtement cutané en regard du QSE. L´échographie mammaire trouvait une plage hypo échogène hétérogène intéressant le quadrant supéro externe (QSE) et la jonction des quadrants supérieurs (JQS) du sein gauche, associée à un épaississement cutané et une adénopathie axillaire d´allure tumorale nécessitant une confrontation histopathologique ainsi qu´une dilatation canalaire rétro aréolaire gauche à surveiller avec un sein droit sans anomalies décelables. La biopsie de cette plage hypo échogène est revenue en faveur d´une mastopathie fibreuse et la cytoponction de l´adénopathie axillaire homolatérale est revenue en faveur d´une lymphadénite de PIRINGER faisant évoquer une toxoplasmose ou mononucléose infectieuse. Un complément de recherche du génome BK par PCR sur la biopsie de l´adénopathie était revenu négatif. Un prélèvement du pus qui s´écoulait à la pression du sein a été fait et est revenu stérile. Un avis en dermatologie a été réalisée pour les lésions des membres inférieurs et une biopsie cutanée des plaques érythémateuses sur les 2 jambes a été effectué et le résultat histologique était en faveur d´une panniculite à prédominance septale avec des granulomes histiocytaires compatibles avec un érythème noueux. Vu l´apparition de nouvelles lésions au niveau du sein gauche et l´absence de diagnostic expliquant le tableau clinique, une échographie mammaire a été refaite et elle décrivait un aspect échographique en faveur de plusieurs collections au sein gauche avec une adénopathie axillaire homolatérale.

Le dossier de la patiente a été ainsi discuté en réunion de concertation pluridisciplinaire et une décision de faire une exérèse chirurgicale de la masse du sein gauche a été prise avec un bilan étiologique plus exhaustif. Une relecture de la biopsie de l´adénopathie axillaire gauche a conclu à une adénite réactionnelle. La patiente a bénéficié d´une tumorectomie du sein gauche au cours de laquelle l´exploration a trouvé du pus enkysté avec une glande mammaire très friable. L´étude histologique de la pièce de tumorectomie trouvait un parenchyme mammaire fait des structures ducto-lobulaires régulières avec un tissu palléal dissocié par des granulomes épithélioïdes et gigantocellulaires centrés par des foyers de nécrose suppurative sans prolifération de tumorale décrivant une mastite granulomateuse avec nécrose suppurée sans prolifération tumorale. Le bilan étiologique était sans particularité. Il était fait de sérologies (VIH, hépatites B, C et de syphilis), de recherche des BK, de recherche des anticorps anticytoplasme des polynucléaires neutrophiles (ANCA), des compléments C3 et C4, d´évaluation de la fonction rénale (urée, créatine et protéinurie) et d´une glycémie à jeun. Le diagnostic final de mastite granulomateuse idiopathique associant un érythème noueux a été retenu.

**Intervention thérapeutique et suivi**: après la réalisation d´une tumorectomie, la patiente a par la suite été mise sous un traitement médical à base de corticoïde par voie orale (prednisone 20mg) pendant 6 semaines et une antibiothérapie à base d´amoxicilline plus acide clavulanique 1g/8 h pendant 21 jours et métronidazole 500mg par 8h pendant 14 jours. La posologie du corticoïde était de 1mg /kg/ jour soit: 60 mg par jour pendant 15 jours, puis 40 mg par jour pendant 15 jours, puis 20 mg par jour pendant 15 autres jours. La patiente bénéficiait d´une surveillance hebdomadaire pendant les 15 premiers jours du traitement puis une surveillance chaque 15 jours jusqu´à l´arrêt du traitement. Au cours de chaque rendez elle bénéficiait d´un examen clinique mammaire évaluant la régression de la symptomatologie. 2 mois après la fin du traitement, on constatait une régression complète de la symptomatologie. L´échographie mammaire de contrôle décrivait une disparition totale des lésions existantes avant le traitement.

**Consentement du patient**: un consentement éclairé de la patiente avait été obtenu.

## Discussion

La MGI représente une maladie inflammatoire bénigne du sein. Il s'agirait d'une maladie idiopathique due à l'influence de certains stimulus environnementaux chez des sujets génétiquement prédisposés [1]. Le mécanisme pathologique reste encore mal connu. À ce jour, trois hypothèses principales ont été évoquées pour expliquer cette maladie: la genèse auto-immune, la maladie infectieuse et les troubles hormonaux [4,5]. Cette pathologie surviendrait essentiellement chez la femme en période d´activité génitale [6] mais peut également survenir chez la femme ménopausée. Dans une étude [7] réalisée sur une série de 20 cas, les auteurs rapportaient que la MGI représentait 2% des pathologies mammaires prises en charge dans leur structure avec une moyenne d´âge de 38,1 ans et 55% des femmes appartenaient à la tranche d´âge de 30 à 39 ans. Sur le plan clinique, le maitre symptôme de la MG est une masse douloureuse et environs 50% des patientes développent un érythème et une tuméfaction en tant que symptômes d'une inflammation du sein atteint. D'autres symptômes sont l'hyperémie, la rétraction aréolaire, la fistule et l'ulcération. Environ 37% des patientes présentent des signes d'abcès [8].

Parfois la MGI peut se manifester par un durcissement du sein avec des collections de pus dans un contexte non fébrile faisant évoquer un abcès froid du sein et entrainant une multitude de consultation et de tentative de prise en charge [3]. Cette symptomatologie reste tout de même non spécifique. A l´imagerie, les lésions décrites ne diffèrent pas de celles décrites dans certaines formes de cancers du sein. Dans notre cas précis, la mammographie décrivait une opacité à contours mal définis et l´échographie mammaire trouvait une plage hypo échogène hétérogène associée à un épaississement cutané ainsi qu´une adénopathie axillaire avec de nombreux foyers de collections. Le diagnostic de certitude est basé sur l´histologie soit d´un fragment de biopsie et/ou d´une pièce de tumorectomie. L´étude histologique de notre cas trouvait un parenchyme mammaire fait des structures ducto-lobulaires régulières avec un tissu palléal dissocié par les granulomes épithélioïdes et gigantocellulaires centrés par des foyers de nécrose suppurative sans prolifération de tumorale. La prise en charge thérapeutique repose essentiellement sur la chirurgie qui consiste en une exérèse large des lésions, précédée d´une corticothérapie visant à réduire les lésions [6].

## Conclusion

La mastite granulomateuse idiopathique est une entité clinique rare qui pose un problème diagnostic comme nous le constatons dans ce cas. Sa symptomatologie et son imagerie restent atypiques et non spécifiques. La biopsie est parfois non satisfaisante et le recours à une exérèse large pour étude histologique est plus judicieux en cas de résultat biopsique non concordant avec la symptomatologie.

## References

[1] Maione C, Palumbo VD, Maffongelli A, Damiano G, Buscemi S, Spinelli G (2019). Diagnostic techniques and multidisciplinary approach in idiopathic granulomatous mastitis: a revision of the literature. Acta Biomed.

[2] Fahmy J, Halabi-Tawil M, Bagot M, Tournant B, Petit A (2015). Érythème noueux au cours d´une mastite granulomateuse idiopathique. Ann Dermatol Venereol.

[3] Conte AB, Nyingone S, Jayi S, Soule HM, Alaoui Fdili FZ, Chaara H (2020). Therapeutic and diagnostic challenge of idiopathic granulomatous mastitis: a case report and review of the literature.

[4] Sheybani F, Sarvghad MR, Naderi HR, Gharib M (2015). Treatment for and clinical characteristics of granulomatous mastitis. Obstet Gynecol.

[5] Hello M, Néel A, Graveleau J, Masseau A, Agard C, Caillon J (2013). La mastite granulomateuse idiopathique. Rev Med Interne.

[6] Boufettal H, Mahdaoui S, Noun M, Hermas S, Samouh N, Benayad S (2011). Mastite granulomateuse idiopathique d´évolution favorable sous traitement médical. Rev Med Interne.

[7] Boufettal H, Essodegui F, Noun M, Hermas S, Samouh N (2012). Mastites granulomateuses idiopathiques : à propos de vingt cas. J Radiol Diagnostique Interv.

[8] Freeman CM, Xia BT, Wilson GC, Lewis JD, Khan S, Lee SJ (2017). Idiopathic granulomatous mastitis: a diagnostic and therapeutic challenge. Am J Surg.

